# Supplementary material for: Seminoma and Embryonal Carcinoma Footprints Identified by Analysis of Integrated Genome-Wide Epigenetic and Expression Profiles of Germ Cell Cancer Cell Lines
Source: PLoS One. 2014 Jun 2;9(6):e98330. doi: 10.1371/journal.pone.0098330 (PMC4041891; doi:10.1371/journal.pone.0098330)
Supplement: File S1 — ZIP file containing DMRforPairs output for significant regions. Please start from the html files. (ZIP) [file pone.0098330.s008.zip › figures/22151.pdf]

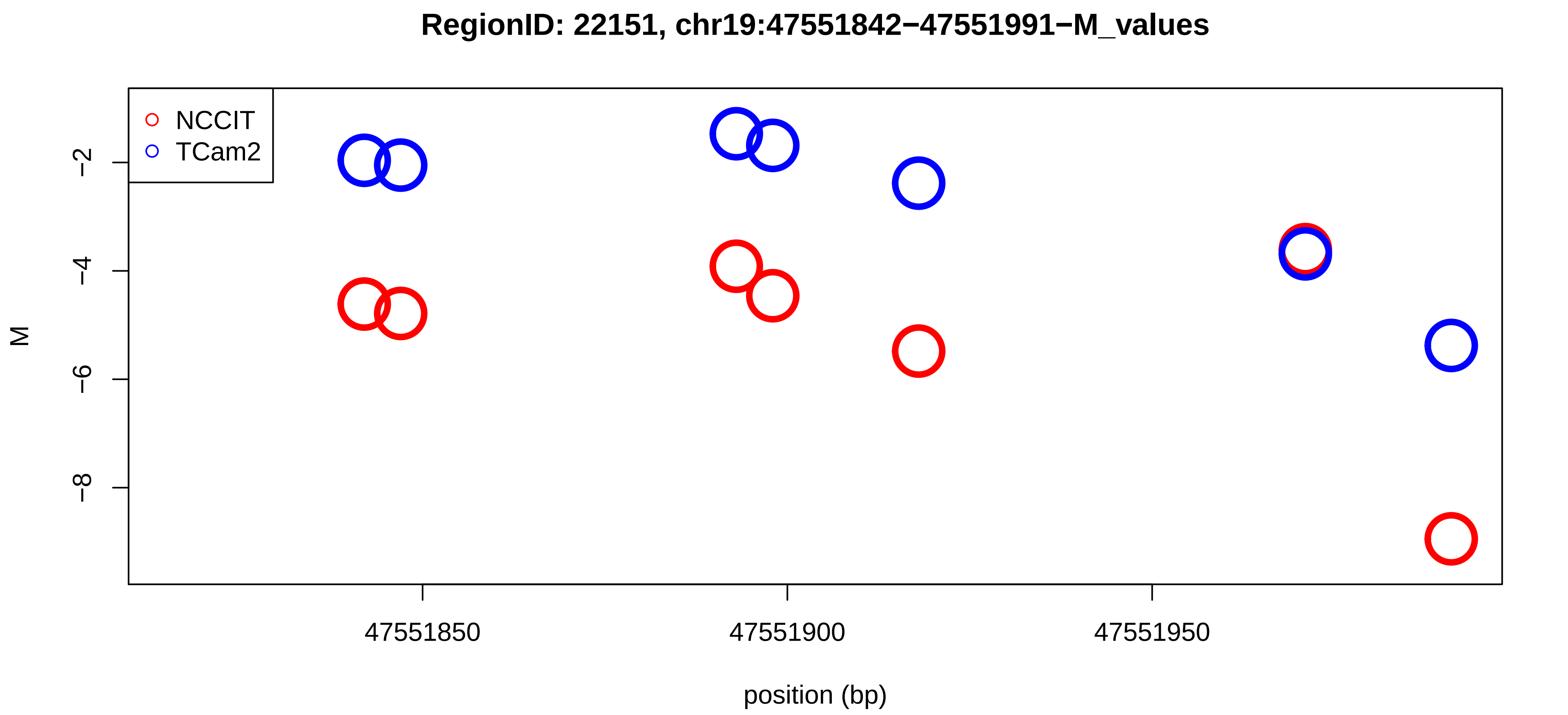

RegionID: 22151, chr19:47551842−47551991−M_values
NCCIT
TCam2
M
−2
−4
−6
−8
47551850
47551900
47551950
position (bp)

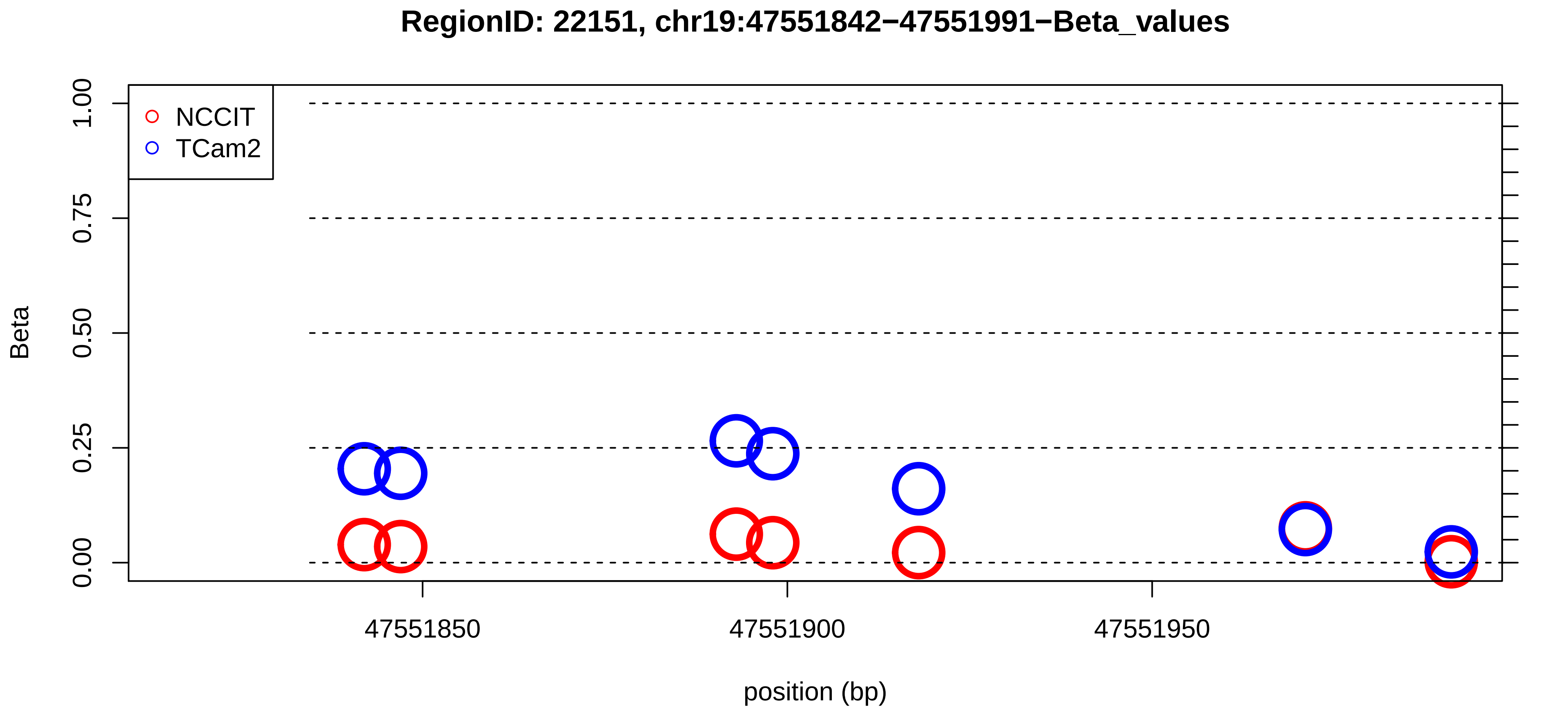

RegionID: 22151, chr19:47551842−47551991−Beta_values
NCCIT
TCam2
Beta
0.00
0.25
0.50
0.75
1.00
47551850
47551900
47551950
position (bp)
